# Cytocompatibility of a conductive nanofibrous carbon nanotube/poly (L-Lactic acid) composite scaffold intended for nerve tissue engineering

**DOI:** 10.17179/excli2015-282

**Published:** 2015-07-27

**Authors:** Mahboubeh Kabiri, Saeed Oraee-Yazdani, Masumeh Dodel, Hana Hanaee-Ahvaz, Sara Soudi, Ehsan Seyedjafari, Mohammad Salehi, Masoud Soleimani

**Affiliations:** 1 Department of Biotechnology, College of Science, University of Tehran, Tehran, Iran; 2 Department of Stem Cell Biology, Stem Cell Technology Research Center, Tehran, Iran; 3 Department of Nanotechnology and Tissue Engineering, Stem Cell Technology Research Center, Tehran, Iran; 4 Functional Neurosurgery Research Center, Shohada Tajrish Hospital, Shahid Beheshti University of Medical Sciences, Tehran, Iran; 5 Department of Textile Engineering, Amirkabir University of Technology, Tehran, Iran, Stem Cell Technology Research Center, Tehran, Iran; 6 Department of Immunology, Faculty of Medical Science, Tarbiat Modares University, Tehran, Iran; 7 Department of Biotechnology, Faculty of Medicine, Shahid Beheshti University of Medical Sciences, Tehran, Iran; 8 Department of Hematology, Faculty of Medical Science, Tarbiat Modares University, Tehran, Iran

**Keywords:** Nerve tissue engineering, olfactory ensheathing cells, carbon nanotube, composite scaffold, electrospun nanofiber

## Abstract

The purpose of this study was to fabricate a conductive aligned nanofibrous substrate and evaluate its suitability and cytocompatibility with neural cells for nerve tissue engineering purposes. In order to reach these goals, we first used electrospinning to fabricate single-walled carbon-nanotube (SWCNT) incorporated poly(L-lactic acid) (PLLA) nanofibrous scaffolds and then assessed its cytocompatibility with olfactory ensheathing glial cells (OEC). The plasma treated scaffolds were characterized using scanning electron microscopy and water contact angle. OECs were isolated from olfactory bulb of GFP Sprague-Dawley rats and characterized using OEC specific markers via immunocytochemistry and flow cytometery. The cytocompatibility of the conductive aligned nano-featured scaffold was assessed using microscopy and MTT assay. We indicate that doping of PLLA polymer with SWCNT can augment the aligned nanosized substrate with conductivity, making it favorable for nerve tissue engineering. Our results demonstrated that SWCNT/PLLA composite scaffold promote the adhesion, growth, survival and proliferation of OEC. Regarding the ideal physical, topographical and electrical properties of the scaffold and the neurotrophic and migratory features of the OECs, we suggest this scaffold and the cell/scaffold construct as a promising platform for cell delivery to neural defects in nerve tissue engineering approaches.

## Introduction

Cell therapy has a promising potential for the treatment of neurodegenerative diseases or trauma-related injuries that are otherwise very difficult to cure by conventional approaches. Damages to nervous tissues, including spinal cord, brain and peripheral nerves, can cause debilitating diseases as they usually lead to progressive functional damages. Current approaches basically rely on reduction or inhibition of gliosis by using steroids (Hall and Springer, 2004[10]), immunemodulatory drugs (Bowes and Yip, 2014[4]) or administration of chondroitinase (Bradbury et al., 2002[5]), aiming to minimize secondary complications associated with the injury (Thuret et al., 2006[31]). With the advent of cell-based therapies, the therapeutic potential of different cell types was investigated for nerve regeneration (Hedayatpour et al., 2013[11]). Stem cells from different sources such as bone marrow (BM) (Hafizi et al., 2015[9]), blastocysts (ESC, embryonic stem cells), fetal tissues and neural stem cells have been investigated frequently, with each displaying varying degrees of regeneration and functional restoration. The transplanted stem cell derived neural or glial cells/progenitors can partially reestablish the neural connections and result in recovery of function (Yazdani et al., 2012[37]). 

Schwann cells (SC), the glial cells that ensheath and myelinate axons in peripheral nervous system (PNS), are of most studied cell types that can repair injuries of central nervous system (CNS) owing to their capability to produce neurotropic factors and cell adhesion molecules (Oudega and Xu, 2006[22]). SC are available autologously by a biopsy from a peripheral nerve in the leg. The released neurotrophics can induce repair and survival of the damaged neurons and attract endogenous stem or glial cells to the lesion site (Ogawa et al., 2002[21]). Another cell type that shares many characteristics and even developmental heritage with SC is olfactory ensheathing glial cell (OEC). Normally, their main role in the olfactory system is to facilitate and guide olfactory axons to the hostile olfactory bulb throughout the life ( Ramón-Cueto and Valverde, 1995[26]). They have shown to express different neurotrophic factors including glial derived neurotrophic factor (GDNF), nerve growth factors (NGF), brain-derived neurotrophic factor (BDNF) and their receptors (Woodhall et al., 2001[35]), and can myelinate axons indistinguishably from myelination by Schwann cells (Barraud et al., 2010[2]). The neurotrophic factors expressed and produced by OEC could endow these cells with axon regeneration and remyelination potential over several millimeters of axons (Imaizumi et al., 1998[12]) and ultimately restoration of functional synapses (Barnett et al., 2000[1]). Besides, originating from the neural crest, OEC persist in adult tissues and can be regarded as an abundant and accessible source of autologous cells throughout the life (Barraud et al., 2010[2]). Regarding the availability of patient specific OEC and their neurotrophic characteristics, these cells can be considered as great candidates for cell-based therapy of neural injuries. 

A wide range of bioengineered scaffolds have been developed to assist in the improvement of neural regeneration. An ideal synthetic scaffold must have chemical, mechanical, morphological and electrical properties mimicking the natural ECM of the desired neural tissue. Poly(α-hydroxy esters), such as poly(L-lactic acid) (PLLA) and its breakdown products are shown to be biocompatible with neural cells and tissues both *in* *vitro *and* in vivo *(Schmidt and Leach, 2003). Their tunable degradation rate, the non-immunogenicity and FDA approval has made them enormously attractive in tissue engineering approaches (Potter et al., 2008). Electrospinning these polymers allows for the generation of aligned fibers with diameters in the nano-meter range that are suitable in directed axonal outgrowth through provision of appropriate contact guidance (Chew et al., 2006[6]; Kabiri et al., 2012[14]; Potter et al., 2008[24]). Furthermore, the electrospun nanofibrous sheets have the capacity to be rolled and packed within a defined volume, providing enough substrate for cell transplantation (Goto et al., 2010[8]). 

 We hypothesized that further functionalizing of the PLLA nanofibers with an electrically conductive compound can aid to mimic the inherently conductive nature of the nerve tissues. Electrically conductive materials such as polypyrrole, polyaniline and carbon nanotubes (CNT) have been effectively used in drug delivery and biosensor applications and for the fabrication of nerve guidance conduits in nerve tissue engineering (Chronakis et al., 2006[7]; MacDonald et al., 2008[18]; Schmidt et al., 1997[28]; Voge et al., 2008[33]; Zhang et al., 2007[38]). The resultant conductive composite would inherit both the physical properties of polymeric materials and the electrical characteristics of the conductive material needed for specific applications such as nerve and cardiac tissue engineering.

In the present study we made a conductive nano-featured substrate of single walled carbon nanotubes (SWCNT) and PLLA and assessed its biocompatibility with well characterized OEC, aiming to use the composite system as a nerve guidance conduit and a cell delivery platform to the nerve lesion for nerve tissue engineering purposes. 

## Materials and Methods

### Scaffold fabrication and characterization

Electrospinning (Nanoazma, Iran) was used to construct SWCNT/PLLA aligned nanofibrous scaffolds. PLLA (M_W_=157000, Sigma-Aldrich) was dissolved in a solvent mixture of chloroform and N,N-dimethylformamide (DMF) (8.5:1.5, v/v) to have a final concentration of 3.5 % w/v. SWCNT (Plasmachem) nanoparticles were first well dispersed in chloroform to form a homogenous suspension, and then combined with DMF and PLLA in the same proportions stated above. The final concentration of SWCNT in solution was equivalent to 3 % of the PLLA mass. The polymer solution was ultra-sonicated and stir homogenized overnight before electrospinning. A syringe pump was used to feed the solution through an extension tube ended in a blunted 21-gauge needle. A voltage potential of 25 kV was applied between the needle and the collector. The nanofiber jet was collected on a stainless steel cylinder rotating at 2400 RPM at a fixed distance of 15 cm from the spinneret tip. Oxygen plasma surface treatment was performed using a low frequency plasma generator set on 40 kHz (Diener Electronics). The hydrophobic/hydrophilic nature of the nanofiber scaffolds before and after plasma treatment was evaluated by measuring the contact angle of water droplets using the sessile drop method (G10 contact angle goniometer, Kruss). 

The morphology of the nanofibrous scaffolds and the surface characteristics of cell seeded scaffolds were evaluated by SEM (Philips Xl-30). The scaffold or cell/scaffold constructs were washed thoroughly with PBS and fixed with 2.5 % glutaraldehyde solution for two hours. The dehydrated samples were sputter coated with gold for 90 s and deposited onto SEM stubs. The electrospun nanofibers were imaged and the fiber diameter was measured from 10 random images using Image J software (NIH). 

### OEC isolation and culture

OEC were obtained from olfactory bulbs of adult GFP rats following a procedure approved by the Animal Care and Ethics of Stem Cell Technology research center, Iran. The olfactory bulb was dissected, finely minced and digested in collagenase/dispase II solution as previously described in details (Salehi et al., 2009[27]). After enzyme deactivation the tissue was mechanically triturated and cells were spun down with centrifugation. The cell pellet was cultured in DMEM/ F12 (Gibco) supplemented with 10 % fetal bovine serum (FBS, Sigma), 2 mM L-glutamine (Gibco), 100 IU/ml penicillin and 100 µg/ml streptomycin (Gibco). Cultures were incubated in 5 % CO2 incubator for 18 hours. Next the supernatant was cultured for another 36 hours in a new uncoated flask. The supernatant was then used to feed poly-L-lysine (Sigma) coated flasks, knowing most of the OEC have not attached to the previous uncoated flasks. Cells were cultured on poly-l-lysine coated plates until appropriate cell number was achieved.

### Cell seeding and proliferation assay

Scaffolds were sterilized in 70 % ethanol for 2 hours and then rinsed copiously with PBS prior to use in culture. The biocompatibility of composite scaffolds was assessed by examining cell proliferation. Cell proliferation was measured on day 1, 7, 14 and 21 from a starting cell seeding number of 5000 cells on the SWCNT/PLLA scaffolds, or in a standard poly-l-lysine coated tissue culture polystyrene (TCP) well control. Relative cell number was quantified using the MTT (methyl thiazolyl diphenyl tetrazolium bromide) assay, as per manufacturer’s instructions and compared to 2-dimensional (2D) TCP controls. Briefly, the MTT assay was performed by incubating cells at various time points with MTT (1 mg/ml) solution for 3 hours. MTT absorbance was measured spectrophotometrically at 570 nm and the absorbance was assumed to be directly proportional to the number of viable cells. 

To investigate cell morphology of the OEC on aligned nanofibers, cell-scaffold constructs were fixed using 2.5 % glutaraldehyde solution. The specimens were then undergone dehydration using series of graded ethanol solutions. The dried samples were then sputter coated with gold and observed using SEM. 

Flow cytometery analysis

The enriched cell population was immunostained with cell specific antibodies to determine purity of the culture. In brief, cells were permeabilized with 0.5 % Triton X-100 and blocked with 1 % BSA. Next the cells were stained with anti-P75 (Sigma) and anti-S100 (Chemicon) antibodies. PE-conjugated secondary antibody (Abcam) was used to label the cells. Paraformaldehyde (PFA) fixed cells were analyzed on a FACS Calibur cytometer (Becton Dickinson, San Jose, CA) and analyzed with Win MDI 2.8 software.

### Immunofluerescence assay

A monolayer culture of OEC was fixed with 4 % PFA, and permeabilized with 0.1 % Triton X-100 for intracellular antigen staining, and blocked with 5 % goat serum. Cells were then incubated at 4 °C overnight with anit-P75 primary antibody. Cells were then incubated with PE-conjugated secondary antibody for 3 hours at room temperature. The cells nuclei were stained with 4',6-diamidino-2-phenylindole dihydrochloride (DAPI; 1:1000) and visualized by fluorescent microscope (TE2000-S; Nikon-Eclipse, Tokyo, Japan).

### Statistical analysis

In all the experiments of this original research article the statistical analysis was performed using the unpaired Student’s t test, where results were considered significant when p < 0.05. All experiments were done in triplicate, unless otherwise stated and data expressed as the mean ± standard deviation.

## Results

### OEC characterization

**Figure 1:**
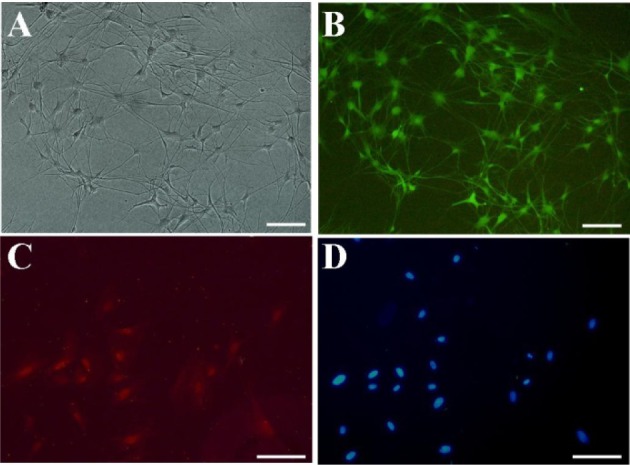
Characterization of isolated OEC. (A) *in vitro* morphology of isolated cells with light microscopy; (B) GFP expressing OEC: The cells show a bipolar or three polar characteristic mostly connected with each other; (C) P75 positive OEC; (D) Nuclei stained with DAPI; Scale bars 10µm.

**Figure 2:**
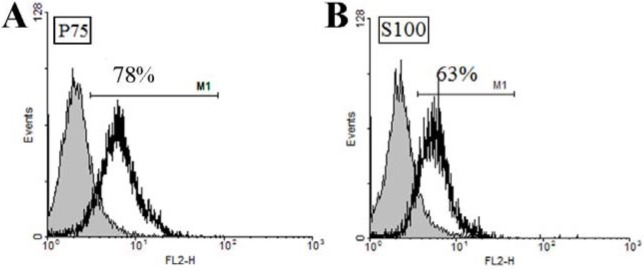
Characterization of isolated OEC. *In vitro* expression of OEC markers as quantified by flow cytometery (A) P75 and (B) S100 markers.

OEC were isolated from olfactory bulb of GFP Sprague -Dawley rats. The isolated cells were phenotypically characterized by flow-cytometery and immune-fluerescence assay before use. The morphology of the cells is shown in Figures 1A and B(Fig. 1). OEC populations are composed of adherent cells with heterogeneous morphologies, but mostly display spindle or polygonal shape. P75 is a neurothrophin receptor (also known as P75NTR, or CD271) expressed on the cell surface of OEC which is normally used for both OEC identification and enrichment. The results of this study indicated that the enriched OEC population was immunopositive for P75 (Figures 1C, D(Fig. 1)). The flow-cytometric results revealed the purity of the cells to be ~78 % (Figure 2A(Fig. 2)). Given that OEC are in fact a glial cell type (also known as olfactory ensheathing glia) residing the olfactory system, expression of glial markers such as glial fibrillary acidic protein and S100 can be used for immunostaining of these cells (Kawaja et al., 2009[15]). Although S100 is basically known as Schwann cells’ marker, but OEC and Schwann cells share many phenotypic and antigenic characteristics. Previous reports also show a close relationship in growth behavior and antigenic markers of OEC and Schwann cells (Ramón-Cueto and Avila, 1998[25]). The isolated cell population used in this study was 63 % immunoreactive to S100 marker (Figure 2B(Fig. 2)).

### Effect of guidance cues on the alignment of OEC

**Figure 3:**
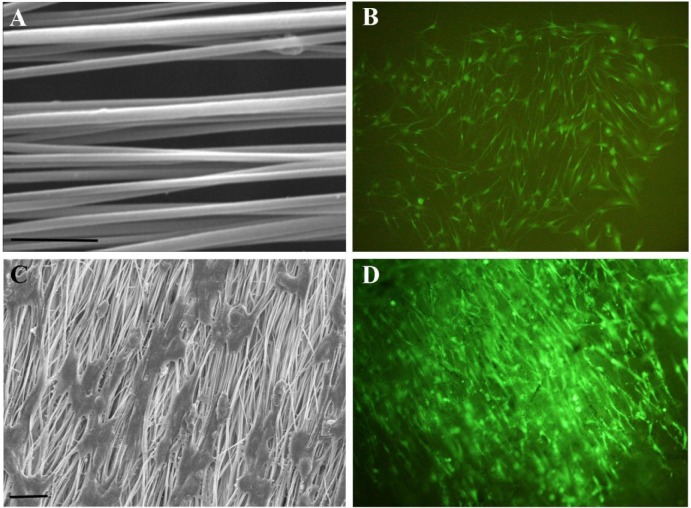
Effect of guidance cues on the alignement of OEC. (A) Aligned SWCNT/PLLA nanofibers used as the substratum for OEC, scale bar= 10µm; (B) OEC grown on culture plates showing random orientation, magnification 100x; (C) SEM micrographs of OEC aligned on nanofiber SWCNT/PLLA scaffolds, scale bar= 2µm; (D) Fluorescence image of aligned OEC grown on SWCNT/PLLA nanofibrous scaffolds, magnification 100x.

The nanostructure of SWCNT doped PLLA scaffold is shown in Figure 3A(Fig. 3). These nanofibers have a narrow range of diameter distribution with an average of ~430nm.We observed an excellent increase in hydrophilicity of the nanofibers after modification with oxygen plasma, with a reduction in water contact angle from 137 to a non-detectable amount. Indeed such surface modifications are commonly used to enhance protein adsorption and subsequent cell attachment onto the scaffolds. We have previously shown that addition of 3 % w/w CNT to the PLLA fibers can dramatically enhance its electrical conductive properties with minimal CNT agglomeration in the fiber constructs and no cytotoxic effects on cellular behavior (Kabiri et al., 2012[14]). The composite scaffolds used in this study were shown to have nanometer diameter fibers, well aligned orientation and high conductivity, totally making them a suitable substratum for nerve tissue engineering applications. The attachment of OEC onto the nanofibrous scaffolds provides a qualitative assessment of the biocompatibility of the composite fibers. When cultured on aligned nanofibers, OEC proliferate and follow the orientation of the fibers; in contrast, on tissue culture plates cells they tend to grow in a random manner (Figures 3B-D(Fig. 3)). As shown in SEM micrographs, OEC tightly attached and got aligned with fibers. Our results indicate that the aligned nanofiber composites can serve as contact guidance to direct cell alignment. This strategy can have promising implications in guided peripheral nerve regeneration.

### Effect of SWCNT/PLLA substrate on OEC proliferation

**Figure 4:**
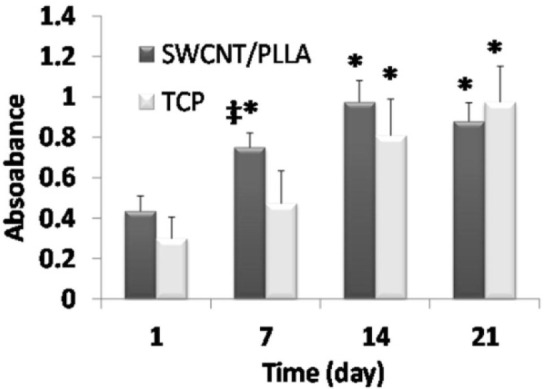
OEC proliferation estimated by MTT assay method at four different time points (average ± standard deviation (SD)). Cells were cultured on the composite scaffolds or TCP plates with the same surface area. The results have been reproduced in two independent studies. Each bar is the average of three replicates; error bars indicate standard deviations. The asterisks imply statistical difference with the first time point (p < 0.05). ‡ implies significant difference with TCP surface at the same time point.

For determining OEC proliferation (expressed by absorbance values), cells were seeded on the scaffolds and subjected to MTT assay at different time points (1, 7, 4, 21 days). The assay indicated that the proliferation of scaffold seeded OEC was just slightly better after 7 days of culture compared to TCP plate cultured cells. However, after 14 and 21 days, there was no statistical difference between cell viability and proliferation on SWCNT/PLLA scaffolds and TCP plates (Figure 4(Fig. 4)). This suggests that SWCNT/PLLA fibers can show OEC supportive capacity just as TCP surfaces. Collectively, the results from Figures 3(Fig. 3) and 4(Fig. 4) suggest good biocompatibility of OEC with CNT/PLLA materials *in vitro*.

## Discussion

Damages to the nervous system leave a significant impact on the quality of life. Amongst neural repair strategies, cell therapy has gained increasing attention due to its potential to replace lost neurons or neuroglias, reduce cysts and cavities and ultimately creating a permissive environment for axon regeneration. Clearly, cell therapy has great potential for repairing damages to the nervous system, however regarding the multifactorial and multiphasic pathophysiology of the brain and spinal cord injury, it needs to be combined with other interventions to maximize tissue regeneration. One strategy is to use scaffolds to aid replacing lost tissue, inhibiting gliosis, enhancing synaptic plasticity and provision of contact guidance for the newly sprouted axons. The promises of bioengineering approaches utilizing combined cellular therapy strategies and biomaterial design and fabrication, provoked us to make a conductive nanofibrous scaffold ideal for nerve tissue engineering and investigate its cytocompatibility with a promising type of cell to promote repair of the injured spinal cord. 

In most cell transplantation approaches, a cell suspension of several hundred thousand is directly injected via fine needles or glass capillaries. However, cell viability and efficacy of engraftments is usually very low (Paul et al., 2009[23]). In recent years, OEC have gained enormous acceptance as a potential source of therapeutic cell in neural lesions and they have shown to promote the survival of injured neurons and functional recovery (Silva et al., 2014[30]). These cells have a dual nature of astroglial cells and Schwann cells (Vincent et al., 2005[32]). Use of OEC is especially attractive given the potential for autologous transplantation, the ability for *in vitro* expansion and excellent neurotrophic and migratory characteristics (Ramón-Cueto and Valverde, 1995[26]). Numerous studies of autologous OEC transplantation to the patients with SCI have proved these cells to be feasible and safe with no evidence of any adverse effects (Mackay-Sim et al., 2008[19]). Particularly, of significant importance for the treatment of SCI is to stimulate the proliferative activity of endogenous neuro progenitor/stem cells along with the migration of resident glial cells to the site of injury that could be best addressed using OEC’s migratory and neurotrophic potential. The transplantation of OEC for SCI is interesting from a translational perspective; however, scaffolds as cell delivery vehicles can be employed to improve cell viability after transplantation (Bensaïd et al., 2003[3]; Malafaya et al., 2007[20]). OEC engraftment on a biocompatible scaffold not only guarantees direct cell delivery to the site of injury but can provide the lesion site with accumulated neural growth factors and establishment of a gradient towards the graft (Kemp et al., 2008[16]). Besides, such scaffold constructs can provide transplanted cells with a suitable microenvironment to maintain their functional phenotype, morphology, cell signaling and guiding potential while avoiding or diminishing immunological rejection of the transplanted cells (Wang et al., 2007[34]).

Having a high aspect ratio, electrospun nanofibers can provide more available surface area for the cells to grow as compared to TCP plates; this in turn enables having more confluent population (Figures 2B, D(Fig. 2)) which means transplanting greater number of cells into the defect site. Besides, the capacity of such small diameter electrospun sheets to be rolled and packed into a cylindrical shape, not only provides an aligned nanosized substratum to direct axons via topographical cues, but also offers ample substrate to deliver repair promoting cells to the site of injury (Li and Shi, 2007[17]). In a similar study, Goto et al. (2010[8]) showed the cytocompatibility of a rolled sheet of collagen scaffold with SCs and proved the efficiency of the construct to promote axonal outgrowth.

Electrical stimulation has previously been shown to guide axon orientation and direct neurite extension (Zhang et al., 2007[38]), outlining the importance of the conductive substrate in enhanced nerve regeneration. Aligned nanofibers of SWCNT-doped PLLA composites have a conductivity of 6 mS/cm which is in sharp contrast to non-conductive PLLA nanofibers (10^-8^–10^-9^ mS/cm) (Kabiri et al., 2012[14]). Doping of PLLA with a conductive substance not only endows the scaffold with conductive properties but can lead to relative reduction in fiber diameter, likely due to higher electrostatic repulsive force to surface tension ratio in the fluid jet of the conductive polymer mixture.

 Only a few studies have addressed the interactions of neural or glial cells with nanofibrous composite substrates (Yang et al., 2005[36]). We had previously shown that CNT/PLLA nanofibrous scaffolds can both support cell adhesion and proliferation and promote neural differentiation of embryonic stem cells (Kabiri et al., 2012[14]). The composite scaffold was indeed able to direct the alignment of sprouted cells from the embryoid bodies. In the present study we observed similar directionality and contact guidance with OEC seeded on SWCNT/PLLA scaffolds. Since this scaffold was destined to serve as a nerve guidance conduit and a cell delivery platform, it was necessary to test its biocompatibility with the desired cell type beforehand. As such, the biological characteristics and proliferation capability of rat OEC were assessed in advance of implementing *in vivo* studies. The viability and/or proliferation of the cells, measured by MTT assay, were similar and even significantly higher on SWCNT/PLLA nanofibers after seven days, as compared to TCP surfaces (Figure 2(Fig. 2)). There was an increasing trend in cell number with time up to 14 days which then tended into the plateau phase likely because of contact inhibition effects in confluent cultures. These results indicate that the plasma treated electrospun SWCNT/PLLA scaffolds are as suitable of a substrate for OEC as is the widely used poly-lysine coated TCP plates. We concluded that the SWCNT/PLLA nanofibers have no cytotoxic or inhibitory effects on OEC survival and proliferation and can be used as a conductive nerve conduit material to facilitate nerve cell proliferation and axonal extension.

We further proved the regenerative potential of this cell/scaffold construct in an *in vivo* study confirming peripheral nerve regeneration capacity in sciatic nerves in rats (Kabiri et al., 2015[13]). Herein we introduced an aligned nanofibrous composite scaffold with structural and electrical features suitable for nerve tissue engineering and showed its cytocompatibility with OEC as a well proved cell type with neuroprotective characteristics for the repair of nerve and spinal cord injuries. We believe this combined cell-scaffold strategy exhibits superior promoting effects as compared with either intervention on its own.

## Conclusion

OEC are considered one of the most promising cell candidates for the treatment of nerve injuries. They can directly participate in axonal extension and myelination and secrete large number of neurotrophic factors for neural differentiation and maturation. On the other hand the topographical, mechanical and electrical cues to direct axonal and neural network connection can be provided by a well-designed scaffold. In the present study we first isolated and characterized OECs from GFP rats and then investigated contact guidance and *in vitro* cytocompatibility of conductive SWCNT/PLLA aligned nanofibrous scaffolds with OEC in order to assess the scaffold suitability as a nerve guidance conduit for nerve tissue engineering. We showed that OEC well adhere and proliferate on these scaffolds and get aligned along the fibers direction. By adding neurotrophic effects of the OEC to the electrical and topographical contact guidance cues of the SWCNT/PLLA scaffold we can promote axonal outgrowth and glial migration from the nerve into the graft and finally better nerve regeneration.

## Acknowledgement

This study was completely supported by stem cell technology research center and university of Tehran. There is no conflict of interest and no benefit of any kind will be received either directly or indirectly by the author(s).

## Conflict of interest

The authors declare that they have no conflict of interest.

## Notes

Dr Mahboubeh Kabiri (Department of Biotechnology, College of Science, University of Tehran, P.O.Box: 14155-6455, Tehran, Iran, Tel: (98) 21 66491622, Mobile: +98 9122116831; E-mail: mkabiri@ut.ac.ir) and Dr Masoud Soleimani (Hematology Department, Faculty of Medical Science, Tarbiat Modares University, P.O.Box: 14115-111, Tehran, Iran, Tel: (98) 21 88861065-7; Mobile: +98122875993; E-mail: soleim_m@modares.ac.ir) contributed equally as corresponding authors.
